# Additional Roles Reimbursement Scheme uptake, patient satisfaction, and QOF achievement: an ecological study from 2020–2023

**DOI:** 10.3399/BJGP.2024.0083

**Published:** 2024-11-26

**Authors:** Chris Penfold, Jialan Hong, Peter J Edwards, Mavin Kashyap, Chris Salisbury, Ben Bennett, John Macleod, Maria Theresa Redaniel

**Affiliations:** National Institute for Health and Care Research Applied Research Collaboration (NIHR ARC) West, University Hospitals Bristol and Weston NHS Foundation Trust; Population Health Sciences, Bristol Medical School, University of Bristol, Bristol.; National Institute for Health and Care Research Applied Research Collaboration (NIHR ARC) West, University Hospitals Bristol and Weston NHS Foundation Trust; Population Health Sciences, Bristol Medical School, University of Bristol, Bristol.; Centre for Academic Primary Care, Population Health Sciences, Bristol Medical School, University of Bristol, Bristol.; Centre for Academic Primary Care, Population Health Sciences, Bristol Medical School, University of Bristol, Bristol.; Centre for Academic Primary Care, Population Health Sciences, Bristol Medical School, University of Bristol, Bristol.; Health Innovation West of England, Bristol.; NIHR ARC West, University Hospitals Bristol and Weston NHS Foundation Trust, Bristol; Population Health Sciences, Bristol Medical School, University of Bristol, Bristol; Centre for Academic Primary Care, Population Health Sciences, Bristol Medical School, University of Bristol, Bristol.; National Institute for Health and Care Research Applied Research Collaboration (NIHR ARC) West, University Hospitals Bristol and Weston NHS Foundation Trust; Population Health Sciences, Bristol Medical School, University of Bristol, Bristol.

**Keywords:** general practice, patient satisfaction, primary health care, workforce

## Abstract

**Background:**

The Additional Roles Reimbursement Scheme (ARRS) was introduced by NHS England in 2019 alongside primary care networks (PCNs), with the aims of increasing the workforce and improving patient outcomes.

**Aim:**

To describe the uptake of direct patient care (DPC)-ARRS roles and its impact on patients’ experiences.

**Design and setting:**

An ecological study using 2020–2023 PCN and practice workforce data, registered patient characteristics, the General Practice Patient Survey, and the Quality and Outcomes Framework (QOF).

**Method:**

Descriptive statistics with associations were examined using quantile and linear regression.

**Results:**

By March 2023, 17 588 full-time equivalent (FTE) DPC-ARRS roles were commissioned by 1223 PCNs. PCNs with fewer constituent practices had more DPC-ARRS roles per population (*P*<0.001), as did PCNs with more FTE GPs per population (*P* = 0.005). DPC-ARRS commissioning did not vary with age, sex, or deprivation characteristics of practice populations. DPC-ARRS roles were associated with small increases in patient satisfaction (0.8 percentage points increase in patients satisfied per one DPC-ARRS FTE) and perceptions of access (0.7 percentage points increase in patients reporting ‘good’ experience of making an appointment per one DPC-ARRS FTE), but not with overall QOF achievement.

**Conclusion:**

The commissioning of DPC-ARRS roles was associated with small increases in patient satisfaction and perceptions of access, but not with QOF achievement. DPC-ARRS roles were employed in areas with more GPs rather than compensating for a shortage of doctors. Single-practice PCNs commissioned more roles per registered population, which may be advantageous to single-practice PCNs. Further evaluation of the scheme is warranted.

## Introduction

The primary care workforce challenge in England is complex and long standing, with shortfalls of GPs and practice nurses alongside increasing healthcare demands.^1^ In 2019, NHS England established primary care networks (PCNs) to increase the primary care workforce to improve personalised and integrated care.^2^ Commissioned by PCNs, the Additional Roles Reimbursement Scheme (ARRS) expands the role of non-medical practitioners in primary care^3^ to improve care delivery, expedite patient access, mitigate rising demand, and provide an advanced career pathway for non-GP practitioners.^4^^,^^5^ Eligible ARRS roles initially included: social prescribing link workers, clinical pharmacists, first-contact physiotherapists, physician associates, and paramedics.^3^ The eligible roles have been expanded annually to include other direct patient care (DPC) and administrative roles,^6^ and the ARRS budget was £1027 million in 2022/2023.^7^

The broadening of the skill mix in NHS primary care through ARRS is occurring rapidly.^8^ Evaluations of the introductions of PCNs and the implementation of the ARRS identified tensions in implementing both changes simultaneously and highlighted the importance of managerial and operational support for their successful combined delivery.^9^^–^^11^ A further theme has been the potential for the ARRS to exacerbate inequalities through recruitment challenges for areas of high deprivation and their inability to compete financially with wealthier PCNs.^12^ However, analysis of NHS primary care workforce data has found that the introduction of PCNs and the overall commissioning of roles through the ARRS did not exacerbate existing inequalities in clinical staff distribution.^13^

Previously, the introduction of new roles in NHS primary care in England was associated with worse patient satisfaction, increased health service costs, and no effect on overall Quality and Outcomes Framework (QOF) achievement, an indicator of clinical effectiveness.^14^^,^^15^ However, broadening the skill mix helped tackle workforce shortages and freed GP time to allow for longer consultations with patients with complex cases.^15^^–^^17^

**Table:** How this fits in

Primary care networks’ commissioning of non-GP direct patient care roles via the Additional Roles Reimbursement Scheme (ARRS) has expanded rapidly. Previously, increased employment of healthcare associate professionals was associated with worse patient satisfaction and perceptions of access, and no impact on Quality and Outcomes Framework (QOF) achievement, but it was not known if these trends remained after the ARRS implementation. This study found small increases in patient satisfaction and access, but not with QOF achievement. Further evaluation to identify if the observed associations can be attributed to the ARRS roll-out and if this represents value for money is warranted.

The aims of this study were to describe variation in the commissioning of ARRS roles during the first 3 years of the scheme and to determine the impact of this initial phase of the scheme on patients’ experiences of primary care services and on clinical effectiveness. This will inform the implementation of the ARRS and future schemes to broaden the primary care skill mix.

## Method

### Study design

This was an ecological study with outcomes recorded at PCN level (role commissioning) and practice level (patient experiences and clinical effectiveness). Practices and PCNs in the 2020–2023 PCN and GP Workforce datasets, and the 2023 General Practice Patient Survey (GPPS) were included. Practices with no registered patients and those not in PCNs were excluded as they cannot commission roles through the ARRS.

### Data sources

Openly accessible data covering 2020–2023 from the following sources (full details of data sources and quality can be found in Supplementary Information S1) were included:

- PCN workforce — quarterly PCN-level full-time equivalent (FTE) employment for 15 DPC staff roles (as of March 2023) funded through the ARRS^18^ (see Supplementary Table S1 for a full list of included and excluded ARRS roles);
- general practice workforce — quarterly snapshots of FTEs for NHS primary care GPs, nurses, DPC, and administrative staff;^19^
- GPPS — the GPPS, run annually, includes responses from >700 000 patients aged ≥16 years about their experiences of general practices.^20^ GPPS fieldwork takes place between January and April each year;
- QOF — the QOF is a pay-for-performance scheme to assess and reward the quality of care provided by practices,^21^^,^^22^ with results published annually;
- general practice registered and weighted population;^23^
- quarterly publication of the number of patients registered at each general practice in England from each of the smallest administrative geographical areas (lower-layer super output areas [LSOAs]).^23^ Payments to general practices and PCNs are adjusted to create a ‘weighted patient count’ using the Carr-Hill formula to reflect the perceived need of the registered population; and
- Index of Multiple Deprivation (IMD) — the English indices of multiple deprivation measure relative levels of deprivation in the 32 844 LSOAs in England.^24^

### Exposure

The exposure was the FTEs of the DPC-ARRS roles at practice level. The PCN workforce data do not record how ARRS roles are shared across practices within PCNs. In the current study the authors assumed an equal allocation of the FTEs across practices, for example, that one FTE paramedic in a four-practice PCN was shared equally as 0.25 FTE per practice.

### Outcomes

#### Patient experience

Measured as the proportion of patients able to access care and proportion satisfied with their care.^14^ These were derived from two GPPS questions relating to a) patient’s experience of making an appointment (access); and b) their overall experience (satisfaction), using a five-point Likert scale. The responses ‘very good’ and ‘fairly good’ were combined as positive indicators of access or satisfaction. GPPS fieldwork is done January–April each year, so in this study the March 2023 PCN workforce was used to most closely reflect the workforce experienced by GPPS responders.

#### Clinical effectiveness

The total QOF points achieved across all domains as a proportion of the maximum available QOF points was used, captured as a percentage, to indicate clinical effectiveness.

### Covariates

Covariates included were the unweighted number of registered patients, the demographics of the registered practice population (mean age, proportion female, and area-level deprivation), and the FTE of GPs and nurses.

#### Estimation of practice-and PCN-level deprivation

The population-weighted mean IMD rank of practices was calculated as the sum of the IMDs of the LSOAs of registered patients divided by the proportion of the practice population from that LSOA. For PCNs, practices were aggregated within PCNs.

### Statistical analysis

#### Temporal trends of ARRS roles

The cumulative commissioning of ARRS roles per quarter from March 2020 to March 2023 are described. The number of patients, weighted by the Carr-Hill formula, per FTE ARRS roles in March 2023 varied by quintiles of PCN-level characteristics; the mean age of patients, number of patients per FTE GP, proportion female, area-level deprivation, and the number of practices in the PCN are described. Quantile regression was used to test for trends.

#### Association of ARRS FTEs with patient experience and clinical effectiveness

Linear regression models were used to estimate associations of ARRS FTEs with patient satisfaction, access to care, and QOF achievement. Models were adjusted for the unweighted number of registered patients and weighted by the number of responders to the relevant GPPS question (patient experience outcomes). Remaining covariates were included as further adjustments. Two-way interactions between ARRS FTEs and GP FTEs and nurse FTEs were included to determine the complementarity of ARRS roles with GPs and nurses.

R (version 4.2) and the ‘tidyverse’ and ‘quantreg’ packages were used.

#### Sensitivity analyses

Regression models for patient outcomes may indicate reverse causality. The authors therefore repeated the fully adjusted regression models with 2020 GPPS outcomes to account for this potential effect. As it had been assumed ARRS FTEs were allocated equally across practices within PCNs, the analyses were repeated but with ARRS FTEs allocated by unweighted registered practice population.

## Results

In total, 1253 PCNs and 6771 GP practices who had submitted data to NHS Digital for March 2023 were included. Of the 6771 practices, 76 (1.1%) were not part of a PCN and excluded from the study.

### PCN commissioning of ARRS roles

**Figure 1.**
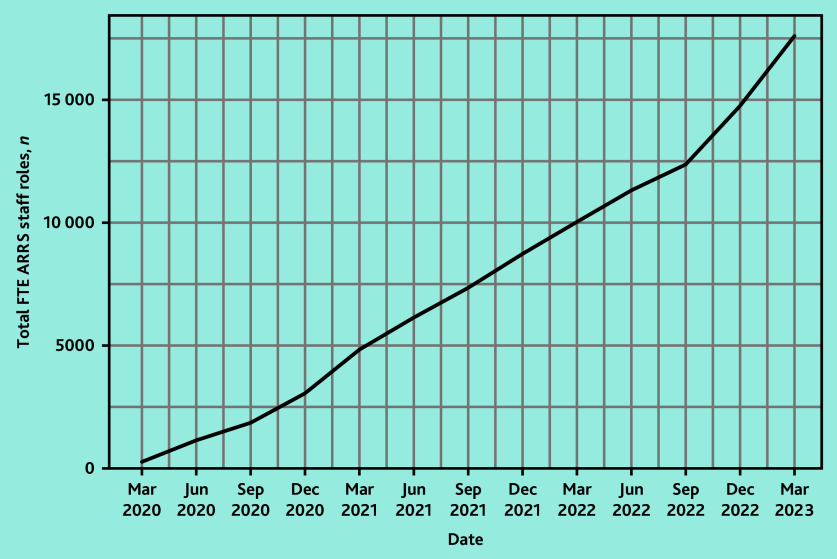
The total FTEs of ARRS-funded staff roles in England between March 2020 and March 2023. ARRS = Additional Roles Reimbursement Scheme. FTE = full-time equivalent.

**Figure 2.**
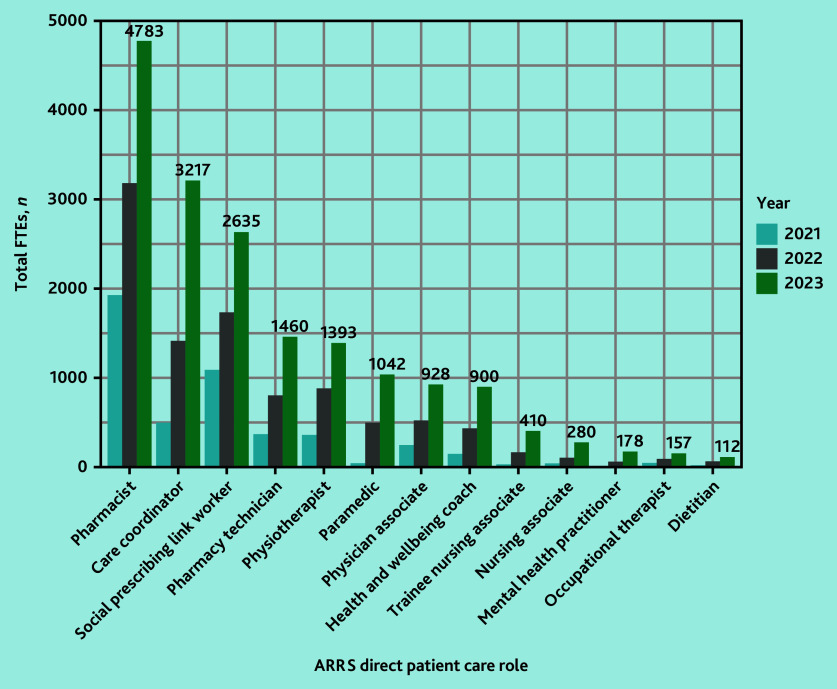
FTEs in DPC-ARRS roles annually 2021–2023, by role. Roles with <100 FTEs in 2023 have been excluded from this plot for clarity. These were (March 2023 FTEs): advanced nurse practitioners (*n* = 47) and podiatrists (*ns* = 45). *N*-value differs from 17 588 because of rounding error as FTE were not whole numbers. ARRS = Additional Roles Reimbursement Scheme. DPC = direct patient care. FTE = full-time equivalent.

In March 2020, 158 of 1253 PCNs (12.6%) reported commissioning a total of 279 FTE staff DPC-ARRS roles (Figure 1). By March 2023, 1223 of 1263 PCNs (96.8%) had commissioned 17 588 FTEs (Figure 2).

The main roles commissioned according to FTEs were pharmacists (*n* = 4783 FTEs, March 2023), care coordinators (*n* = 3217 FTEs), social prescribing link workers (*n* = 2635 FTEs), pharmacy technicians (*n* = 1460 FTEs), and physiotherapists (*n* = 1393 FTEs) (Figure 2 and Supplementary Table S2). The median PCN-level FTEs of ARRS staff roles was 12.6 (interquartile range [IQR] 8.8–17.9) in March 2023 and a median of 2.6 FTE ARRS roles per practice (IQR 1.7–3.8) (data not shown).

### Variation in ARRS commissioning by practice characteristics

**Figure 3.**
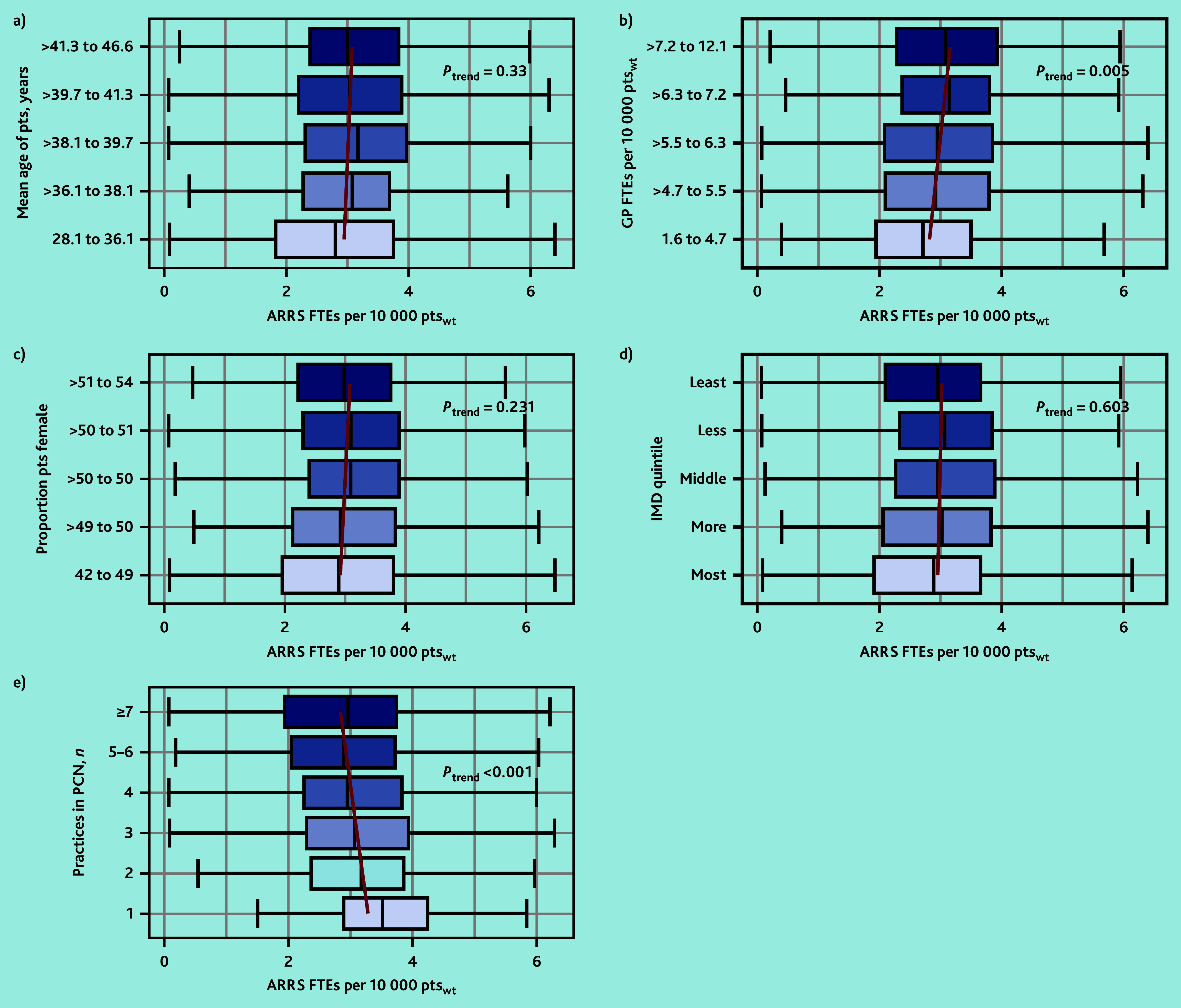
The variation in the PCN-level median ARRS FTEs per weighted number of patients by a) registered patient mean age in years; b) GP FTEs per weighted number of patients; c) proportion of female patients; d) area-level deprivation of registered patients; and e) number of practices in the PCN. Red lines represent the linear trend of median ARRS FTEs per patient. ARRS = Additional Roles Reimbursement Scheme. FTE = full-time equivalent. IMD = Index of Multiple Deprivation. PCN = primary care network. pts = patients. pts_wt_ = weighted patients.

In March 2023, the median PCN-level FTE ARRS roles per 10 000 registered patients (weighted by the Carr-Hill formula) was 2.91 (IQR 2.06–3.80). Figure 3 highlights the variation in the commissioning of ARRS roles by the characteristics of the PCN population. PCNs with the most GP FTEs per 10 000_weighted_ patients had around 0.4 more FTE ARRS roles compared with those with the least GP FTEs (2.73 versus 3.11, least versus most GP FTEs, *P*_trend_ = 0.005). The number of practices within PCNs was negatively associated with the FTEs in ARRS roles per patient. PCNs comprised of one practice compared with the quintile of PCNs comprised of the most practices (7 to 22) had nearly 0.6 more FTEs in ARRS roles per 10 000 patients (3.51 versus 2.95 FTE ARRS/10 000_weighted_ patients, *P*_trend_<0.001). The FTEs in ARRS roles per 10 000 patients varied minimally by mean age of patients (median 2.81 versus 3.00, youngest versus oldest, *P*_trend_ = 0.330), proportion female (2.89 versus 2.98, smallest versus largest proportion, *P*_trend_ = 0.231), or their area-level deprivation (2.92 versus 2.98, most versus least deprived, *P*_trend_ = 0.603).

### Patient outcomes

**Figure 4.**
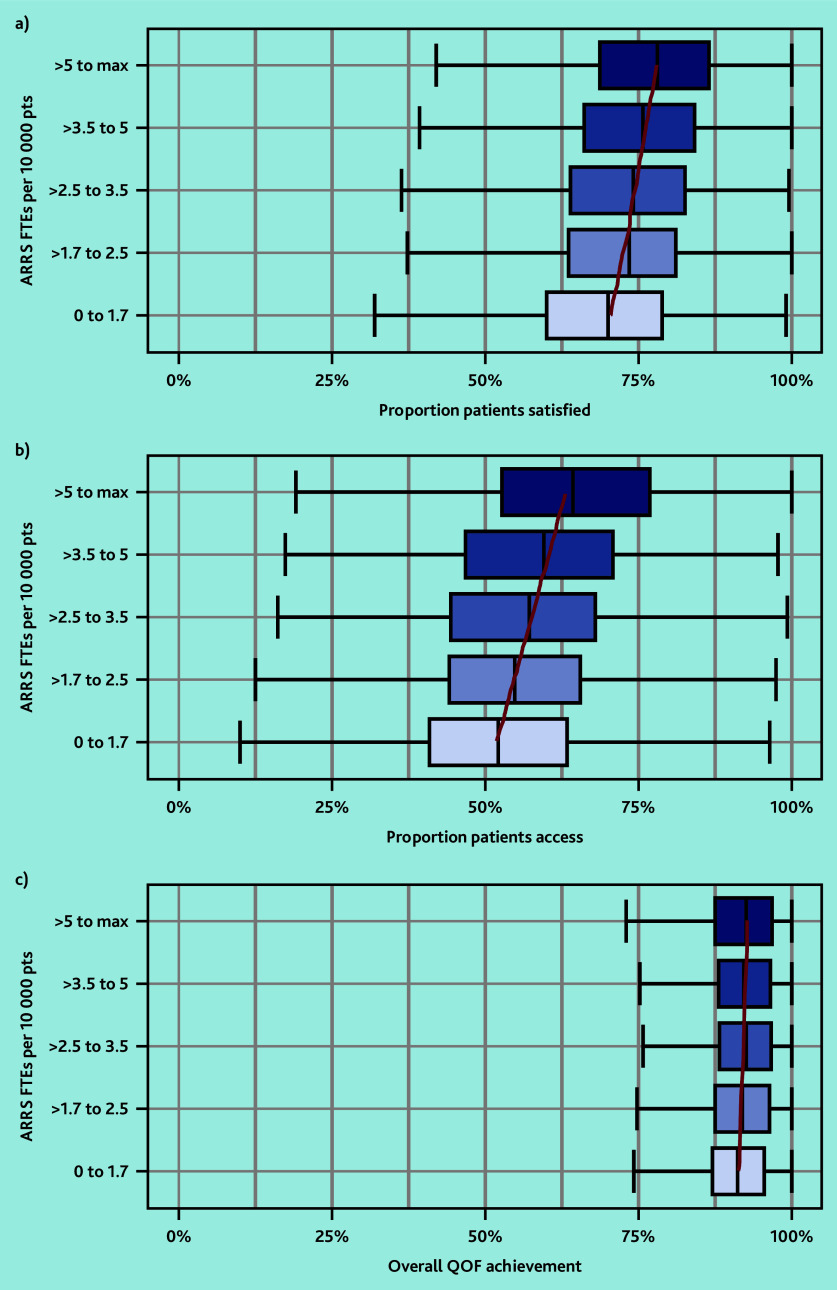
Box plots of a) the practice-level proportion of patients satisfied with their GP service; b) able to make an appointment (access); and c) overall QOF achievement percentage by quintiles of ARRS FTEs per 10 000 patients. Red lines represent the linear trend from quantile regression across exposure categories of median outcome values. ARRS = Additional Roles Reimbursement Scheme. FTE = full-time equivalent. pts = patients. QOF = Quality and Outcomes Framework.

In March 2023, the overall proportion of patients who reported a ‘good’ experience making an appointment at their GP was 54.4% and the proportion satisfied with GP services was 71.3% (data not shown). Practices with more ARRS FTEs per 10 000 patients had a higher proportion of patients satisfied and able to make appointments (Figure 4). The percentage of overall QOF achievement varied minimally by ARRS FTEs. Practices with the most ARRS FTEs (5 to maximum) achieved 1.5 percentage points more of their QOF overall achievement percentage compared with practices with the least ARRS FTEs.

In the adjusted regression models an increase of one FTE in ARRS roles was associated with a 0.80 percentage point increase (95% confidence interval [CI] = 0.60% to 1.01%, *P*<0.001) in the proportion of patients satisfied with their care and a 0.72 percentage point increase (95% CI = 0.46% to 0.97%, *P*<0.001) in the proportion of patients able to make appointments (Table 1). This equates to an increase of approximately 240–400 patients satisfied with their care and 210–350 patients able to make appointments for each FTE in ARRS roles employed in a typical PCN (30 000–50 000 patients). Whereas for overall QOF achievement a one FTE increase in ARRS roles was associated with a 0.04 percentage point decrease in percentage overall achievement, but with CIs extending above the null value (95% CI = −0.21% to 0.12%, *P* = 0.6).

**Table 1.** Linear regression models of ARRS FTEs against the proportion of GPPS responders satisfied with and able to access primary medical care, and QOF overall achievement percentage

**Characteristic**	**Proportion satisfied**			**Proportion able to access services**			**QOF achievement**		

	**Percentage point increase**	**95% CI**	***P*-value**	**Percentage point increase**	**95% CI**	***P*-value**	**Percentage point increase**	**95% CI**	***P*-value**
**Minimally adjusted**									
Total FTEs in ARRS roles	0.44	0.31 to 0.57	<0.001	0.53	0.37 to 0.69	<0.001	0.07	−0.05 to 0.19	0.2
Number of registered patients (100s)	−0.03	−0.04 to −0.03	<0.001	−0.04	−0.05 to −0.04	<0.001	0.00	0.00 to 0.00	>0.9

**Adjusted for all covariates**									
Total FTEs in ARRS roles	0.80	0.60 to 1.01	<0.001	0.72	0.46 to 0.97	<0.001	−0.04	−0.21 to 0.12	0.6
Number of registered patients (100s)	−0.04	−0.05 to −0.03	<0.001	−0.04	−0.04 to −0.03	<0.001	−0.02	−0.02 to −0.01	<0.001
Total GP FTEs	0.65	0.54 to 0.76	<0.001	0.35	0.22 to 0.49	<0.001	0.19	0.09 to 0.28	<0.001
Total nurse FTEs	−0.58	−0.76 to −0.39	<0.001	−0.76	−1.00 to −0.53	<0.001	0.09	−0.08 to 0.26	0.3
Mean patient age, years	0.49	0.39 to 0.59	<0.001	0.49	0.37 to 0.61	<0.001	0.10	0.04 to 0.16	<0.001
Proportion of female patients	0.07	−0.11 to 0.24	0.4	−0.47	−0.69 to −0.24	<0.001	0.37	0.28 to 0.46	<0.001
Population-weighted mean practice deprivation decile	1.05	0.87 to 1.23	<0.001	1.19	0.96 to 1.42	<0.001	0.69	0.57 to 0.81	<0.001
Total FTEs in ARRS roles × total GP FTEs	−0.03	−0.04 to −0.02	<0.001	−0.02	−0.04 to −0.01	0.002	0.01	−0.01 to 0.02	0.3
Total FTEs in ARRS roles × total nurse FTEs	0.01	−0.01 to 0.03	0.2	0.02	0.00 to 0.04	0.10	−0.01	−0.03 to 0.01	0.4

*ARRS = Additional Roles Reimbursement Scheme. FTE = full-time equivalent. GPPS = General Practice Patient Survey. QOF = Quality and Outcomes Framework.*

### Sensitivity analysis

#### Adjustment for 2020 GPPS outcomes

Inclusion of the 2020 GPPS outcomes for satisfaction and access reduced the effect sizes but the CIs still supported the observed positive associations (see Supplementary Table S3).

#### Practice population-weighted deployment of ARRS roles

Changing the assumption made about how ARRS roles were deployed across practices within PCNs from an equal distribution to a registered population-weighted approach reduced the effect size estimates of the regression models, but they still supported the primary analyses (see Supplementary Table S4).

## Discussion

### Summary

The introduction of ARRS had, by March 2023, increased the number of staff in DPC roles by >17 000 FTEs. The commissioning of DPC-ARRS roles does not vary by area-level deprivation. DPC-ARRS roles were employed in areas with more GPs rather than compensating for a shortage of doctors. Single-practice PCNs commissioned more FTE DPC-ARRS roles than PCNs with multiple practices.

### Strengths and limitations

The main strengths are that the findings are representative of the whole of England and that openly available public data were linked. PCN workforce data were initially quite incomplete, but improved rapidly and are now likely to be accurate and reliable. Other strengths are that the study included patient outcomes, the longitudinal nature of the PCN workforce data, and that the patient outcomes used from the GPPS have been used in previous studies,^14^^,^^15^ which facilitates comparisons.

The ecological study design is an important weakness. The authors also do not know how PCNs deployed their ARRS workforce. Two alternative assumptions about how PCNs deployed their ARRS workforce were considered: 1) evenly distributed between their constituent practices, and 2) population-weighted distribution between practices. Neither approach fully accounts for the large variation between PCNs reported from qualitative interviews in how ARRS has been operationalised.^10^ Early in the ARRS the completeness of workforce reporting by PCNs was relatively poor. The current study could not differentiate between PCNs not commissioning any ARRS roles and low engagement with workforce reporting. The current study did not account for when PCNs commissioned each of their ARRS roles, which may have varied by up to 3 years between PCNs, and over this period new roles may have been integrated into practices or PCNs more effectively. The FTEs of staff in ARRS roles is small relative to GPs and therefore most patient contacts, which inform patient experiences of their general practice and hence their responses to the GPPS, will still be with GPs. The response rate for the GPPS is low but its methodology mitigates the effects of various potential sources of bias, including weighting to account for unequal probability of selection, differences between responders and non-responders, and the eligible population characteristics.^25^ Since the QOF is a pay-for-performance scheme, high QOF attainment may reflect a focus on incentivised activities rather than high-quality care.^26^

### Comparison with existing literature

The current study found no variation in the commissioning of DPC-ARRS roles by area-level deprivation, in agreement with previous analysis of PCN workforce data,^13^ but contradictory to findings from qualitative interviews with NHS staff.^12^ These discrepant findings may be because PCNs in more deprived areas are commissioning staff through the scheme, but not the staff they want or need for their population’s needs. Alternatively, as reported elsewhere,^27^^–^^29^ the Carr-Hill adjustment does not sufficiently account for the additional health needs of more deprived populations. A Health Foundation report found that by using the new NHS England PCN-adjusted population rather than the Carr-Hill adjustment, PCNs in more deprived areas had fewer ARRS staff than those in less deprived areas.^30^ This inequality has reduced incrementally between 2020 and 2023, during which time the allocation of PCN funding has improved in more income-deprived areas.^31^

PCNs with fewer practices commissioned more ARRS roles than those with more practices, and this was most pronounced for single-practice PCNs. A possible explanation for the current findings is that PCNs with fewer practices are organisationally simpler, meaning that employing staff through the ARRS is more akin to employing staff directly through the practice. This could incentivise practice mergers, which may be at the expense of patient satisfaction and access, and continuity of care, which are reduced in large practices.^32^^,^^33^ It has been highlighted previously that >40% of PCNs were not of the recommended size and that this may affect their ability to effectively utilise investment.^34^ The current findings support this concern. Additionally, one of the aims of the ARRS is to expedite patient access; however, previous schemes that prioritised access have been at the expense of continuity of care.^35^

Commissioning of ARRS roles followed similar trends to that of GPs. PCNs with more GP FTEs per 10 000 needs-adjusted patients commissioned more DPC-ARRS FTEs. Whether this addresses the needs of the local population or is a further reflection of local staff recruitment challenges is unknown. The potential for this trend to exacerbate existing inequalities in the distribution of the primary care workforce does not align with other research that found this was not the case.^13^

Practices with more FTEs in ARRS roles had slightly better patient-reported satisfaction and ability to make appointments, but the effect size was very small, especially given the investment in the scheme. This contrasts with cross-sectional^15^ and longitudinal^14^ findings using the same GPPS outcomes in 2019 and earlier. These studies were conducted during a period of relative stability before the COVID-19 pandemic whereas the current study may have been affected by restrictions imposed during the pandemic, despite the primary endpoint being in the post-pandemic recovery period. Additionally, the transfer of tasks from GPs to non-GP practitioners was identified as a key challenge to be faced by the newly formed PCNs.^36^ In the current study, the outcome was 4 years after PCNs were introduced, which may have given PCNs sufficient time to address this challenge. The finding of no association between FTEs in ARRS roles and clinical effectiveness, captured as overall QOF achievement, is in agreement with longitudinal^14^ and cross-sectional^15^ findings relating to non-GP and non-nurse health professionals.

### Implications for research and practice

ARRS has expanded existing roles and introduced new roles into NHS primary care in England. It remains unclear if minimal increases in patient perceptions of access and overall care represent value for money. The relationship between PCN organisational structure and commissioning of ARRS roles may have implications for the evolution of PCNs.

Future research could address the lack of patient-level data on consultations with ARRS staff. This would provide a better understanding of the types of patients seen by each of the roles and outcomes of those consultations, which would inform overall workforce planning, training needs of staff in these roles, and opportunities to develop these roles. Finally, it may be beneficial to understand how and why the structure of PCNs affects their ability to commission ARRS roles.

## Data

Data used in this study are publicly available and can be accessed via the URLs in references 18–20, 22, and 23.
